# Crystallinity Influence on Mechanical Properties and Oxidation Resistance of Zirconium Silicon Nitride Thin Films

**DOI:** 10.1021/acsomega.5c06362

**Published:** 2025-12-02

**Authors:** Luís F. S. Sabino, Paula L. L. Araújo, Fábio S. Oliveira, Iago L. Dias, Ronaldo L. Rezende, Igor Z. Damasceno, Roberto Hübler, Eduardo K. Tentardini

**Affiliations:** † Universidade Federal de Sergipe, Av. Marcelo Deda Chagas, São Cristóvão, Sergipe 49107-230, Brasil; ‡ 28123 Universidade Federal do Rio Grande do Norte, Av. Senador Salgado Filho, 3000 Natal, Rio Grande do Norte, Brasil; § 28102 Pontifícia Universidade Católica do Rio Grande do Sul, Av. Ipiranga, 6681 Porto Alegre, Rio Grande do Sul, Brasil

## Abstract

Zirconium silicon nitride thin films containing 8 at. % Si (Zr_39_Si_8_N_53_) were codeposited via reactive magnetron sputtering at room temperature and at high substrate temperatures to produce amorphous and crystalline coatings. The main purpose was to investigate the dominant factor that determines the mechanical and thermal properties for these thin films: the crystallinity or the silicon content. Samples were characterized by RBS, SEM-FEG, GIXRD, nanohardness, and high-temperature oxidation. Crystalline Zr_39_Si_8_N_53_ samples exhibited changes in preference grown orientation and reduction in crystallite size when compared to Zr_46_Si_2_N_52_ samples (used for comparative purposes). These structural modifications caused a hardness value increase superior to 35% when compared to amorphous Zr_39_Si_8_N_53_ samples, confirming the direct influence of crystallization on this mechanical property. All crystalline samples failed under high temperature tests, whereas only amorphous Zr_39_Si_8_N_53_ samples demonstrated resistance to oxidation at 600 °C, indicating that a high silicon content combined with an amorphous structure plays a primary role for improving the oxidation resistance in Zr–Si–N thin films.

## Graphical abstract

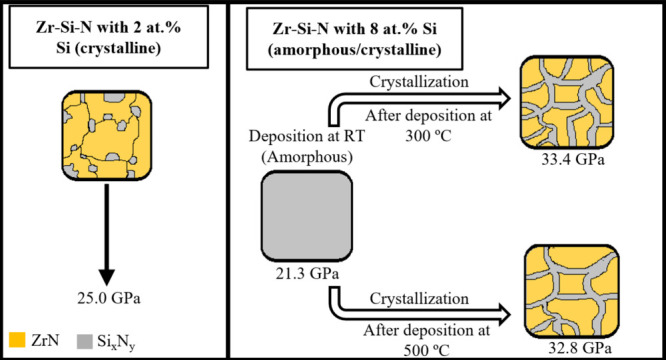

## Introduction

Transition metal nitride thin films have been widely studied as a result of their excellent mechanical properties and thermal stability.^1–3^ In particular, zirconium nitride (ZrN) coatings have become a real possibility in metal-working applications due to high hardness and superior tribological performance. Regardless of the good mechanical properties, the oxidation temperature of ZrN is near to 600 °C, limiting its applicability in high-temperature environments.^4–6^

An effective approach to enhancing the oxidation resistance of ZrN is the incorporation of a third chemical element into its matrix, such as silicon. Zirconium silicon nitride (Zr–Si–N) thin films have gained attention as a promising alternative to pure ZrN coatings, offering superior mechanical and tribological performance, as well as improved oxidation resistance at high temperatures.^7–11^

Previous research^12–15^ has shown that incorporating up to 6 at. % silicon into Zr–Si–N coatings leads to the amorphous silicon nitride (α-Si_3_N_4_) nanoclusters formation at the ZrN grain boundaries, enhancing the mechanical and tribological performance, particularly when Si content is near to 2 at. %. On the other hand, when silicon addition exceeds 6 at. %, significant α-Si_3_N_4_ compound growth is observed, promoting a loss of the thin film crystallinity and reducing the overall coating properties, except for oxidation resistance.^16–18^

An intriguing aspect regarding Zr–Si–N thin films is the correlation between mechanical properties degradation and the amorphous structure presence,^19–21^ raising a question of the primary cause responsible for these losses: the increased silicon concentration in the coatings or the structure amorphization.

Earlier studies^22–26^ have reported alterations in mechanical and tribological properties for nitride, oxide, and carbide coatings due exclusively to the transition from amorphous to crystalline condition. However, as far as we can detect in the literature, there is not a detailed investigation that thoroughly explored this specific topic for Zr–Si–N thin films.

Assuming that the decline in mechanical properties could be predominantly caused by the amorphization phenomenon, Zr–Si–N thin films with Si addition superior to 6 at. %, while maintaining their crystallinity, could potentially exhibit higher hardness values compared to Zr–Si–N coatings with 2 at. % Si added, thereby preserving their performance against high-temperature oxidation.

In this scenario, to elucidate the dominant factor affecting the mechanical and oxidative properties of zirconium silicon nitride films (crystallinity or silicon content), Zr–Si–N coatings containing 8 at. % Si were deposited by reactive magnetron sputtering at room temperature and heating the substrate, to produce amorphous and crystalline thin films, respectively. The samples were characterized by Rutherford backscatter spectroscopy (RBS), grazing incidence angle X-ray diffraction (GIXRD), field emission gun-scanning electron microscopy (SEM-FEG), nanohardness analyses, and oxidation tests.

## Material and Methods

Zr–Si–N samples were deposited using an AJA model Orion 5-HV sputtering system equipped with a rotating sample holder with an angular speed of 10 rpm. Targets of Zr and Si with 99.8% and 99.9% purity were used in direct current (DC) and radio frequency (RF) power supplies, correspondingly.

Polyethylene and silicon wafers were used as substrates based on the characterization technique to be employed: polyethylene for RBS and silicon for GIXRD, nanohardness, microscopy, and oxidation tests. The samples were cleaned in an ultrasonic bath with acetone for 15 min and then inserted into the chamber under a vacuum.

Zirconium silicon nitride thin films with 8 at. % Si addition were produced at room temperature, allowing them to be amorphous, and with heating at 300 and 500 °C, to promote thin film crystallization. These heating temperatures were chosen to preserve the Si_*x*_N_*y*_ grains integrity, avoiding solid solution formation or silicon nitride dissolution, because thermal decomposition for this material is initiated at temperatures exceeding 650 °C.^27^ The substrate heating was conducted by using a 1000 W tungsten halogen lamp positioned behind the sample holder. Additionally, for comparative purposes, Zr–Si–N coatings with 2 at. % Si added were deposited without substrate heating.

For thin film depositions, most parameters were kept constant, except for the substrate temperature and the power applied to the Si target. Ar and N_2_ flow rates were set to 19 and 2 sccm, respectively, and base pressure was maintained at 3 × 10^–5^ Pa with a working pressure of 4 × 10^–1^ Pa, without substrate bias, and a target-substrate distance of 100 mm; the power applied to the Zr target was 120 W, and the powers for the Si target were 16 and 80 W for samples with 2 and 8 at. % Si added, correspondingly.

Deposition times were determined based on the characterization techniques to be used: 15 min for RBS samples and 60 min for other characterizations.

RBS analyses were performed using alpha (He^2+^) particles accelerated to 2.5 MeV in a 3 MV Tandetron particle accelerator. The system features a silicon-based detector positioned at a 165° angle relative to the incident beam, with a resolution of approximately 12 keV. The results obtained were processed using RUMP software.

Microstructural analyses and the phase identification were conducted by GIXRD using a Shimadzu XRD-6000 system (30 kV, 30 mA; Cu Kα radiation, λ = 0.154 nm). The scan was performed with an incidence angle of 1°, step of 0.02°, scan speed of 2°/min, and a 2θ range from 25° to 65°. A Carl Zeiss Auriga 40 SEM-FEG instrument was used for cross-section analyses.

Thin film hardness values were measured using a Fischerscope HV 100 nanohardness tester, equipped with a Berkovich indenter. All indentations were carried out with a maximum depth of 10% of the coating thickness and a maximum load of 5 mN in dynamic load–unload cycles.

The oxidation tests were carried out in an electric resistance furnace, model JUNG LF00612. The test temperature was maintained at 600 °C in ambient air for 30 min, with a heating rate of 10 °C/min. After the tests, samples were analyzed using GIXRD and the surface morphology was examined by SEM with a JEOL JCM 5700 system.

## Results and Discussion

### Composition and Surface Morphology Characterization

Zirconium silicon nitride thin films were deposited at room temperature with different silicon concentrations and analyzed by RBS, aiming to determine the chemical composition for each coating.

The selection of samples for this work was based on Zr–Si–N thin films properties, reported earlier in the literature: 2.0 at. % Si added (sample ZrSiN_2%), where a crystalline structure and superior mechanical properties are expected, and 8.0 at. % Si added (sample ZrSiN_8%), where the structure is expected to be amorphous.

Figure 1a shows the RBS spectra for both samples. Polyethylene substrates were employed to facilitate the quantitative analyses of light chemical elements, such as nitrogen.

**Figure 1:**
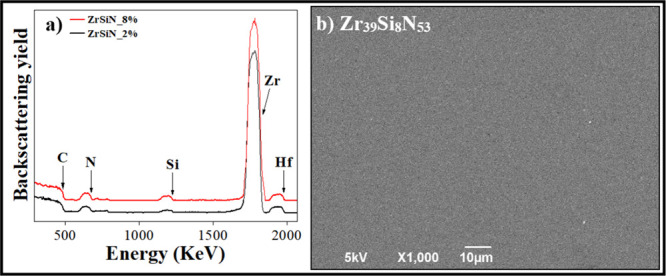
(a) RBS analyses for samples ZrSiN_2% and ZrSiN_8%. (b) SEM image for the as deposited Zr_39_Si_8_N_53_ sample.

In addition to peaks corresponding to carbon from the substrate and zirconium, silicon, and nitrogen from the thin films, a peak associated with hafnium can be identified and is attributed to its occurrence as impurity in the Zr target used during deposition, as previously observed in the literature.^28^ Table 1 summarizes the chemical composition in terms of Zr, Si, and N for samples ZrSiN_2% and ZrSiN_8%.

**Table 1:** Chemical Composition for Zr–Si–N Samples with 2 and 8 at. % Si Added

	Chemical concentration (at. %)		
Samples	Zr	Si	N
ZrSiN_2%	45.7 ± 0.2	2.0 ± 0.3	52.3 ± 0.9
ZrSiN_8%	38.6 ± 0.2	8.0 ± 0.4	53.4 ± 0.9

Based on the RBS results, new zirconium silicon nitride thin films were deposited on silicon substrates. Zr–Si–N samples with 8.0 at. % Si added were deposited at room temperature (sample Zr_39_Si_8_N_53_), at 300 °C (sample Zr_39_Si_8_N_53__300C), and 500 °C (sample Zr_39_Si_8_N_53__500C). Since the only deposition parameter varied was the substrate temperature, it is expected that the chemical compositions of these samples would remain identical. Besides, Zr–Si–N samples with 2.0 at. % Si added were deposited at room temperature (sample Zr_46_Si_2_N_52_) for comparative purposes.

Aiming to verify the samples’ integrity, SEM surface images of all samples were registered. Figure 1b shows the micrograph for sample Zr_39_Si_8_N_53_, and similar images (not shown) were obtained for the other samples. It is possible to observe that this sample is characterized by a homogeneous surface, without cracks, bubbles, or visible defects.

### Structure Analysis

GIXRD patterns from all deposited samples are shown in Figure 2.

**Figure 2:**
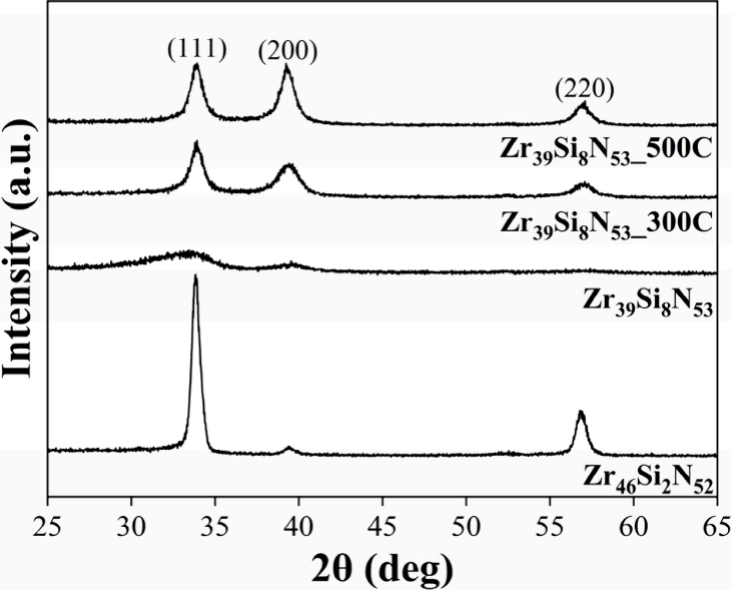
GIXRD patterns for Zr_46_Si_2_N_52_, Zr_39_Si_8_N_53_, Zr_39_Si_8_N_53__300C, and Zr_39_Si_8_N_53__500C samples.

As expected, sample Zr_46_Si_2_N_52_ exhibited crystalline structure, revealing diffraction peaks corresponding to the (111), (200), and (220) planes, which are characteristic from the ZrN rock-salt structure phase (PDF-35-753). On the other hand, sample Zr_39_Si_8_N_53_ exhibited amorphous behavior, also predicted, as a result of the higher Si content in its composition.

As intended, substrate heating at 300 and 500 °C proved to be efficient in increasing atomic mobility, promoting a clear crystallization of the Zr_39_Si_8_N_53__300C and Zr_39_Si_8_N_53__500C samples, as evidenced by the presence of the three diffraction peaks associated with ZrN for both samples. Clearly, due to the higher temperature, sample Zr_39_Si_8_N_53__500C exhibits more well-defined diffraction peaks in comparison to sample Zr_39_Si_8_N_53__300C. Besides, peaks referring to silicon phases could not be identified.

Notably, the addition of 8 at. % Si, combined with the elevated substrate temperatures during deposition, resulted in broader diffraction peaks and an increase of the (200) peak when compared to sample Zr_46_Si_2_N_52_, which presents a preferential growth along the (111) plane.

It is worth mentioning that it is not possible to observe a shift for lower or higher angles of ZrN diffraction peaks among samples Zr_46_Si_2_N_52_, Zr_39_Si_8_N_53__300C, and Zr_39_Si_8_N_53__500C, i.e., there is no variation in interplanar distance for all crystalline samples, suggesting that silicon atoms in the coatings are present always as the Si_*x*_N_*y*_ amorphous phase, without a solid solution formation, regardless of the substrate heating. That is consistent with results obtained in previous studies that reported the coexistence of ZrN and Si_*x*_N_*y*_ compounds in Zr–Si–N thin films, despite the Si content added.^17,29^

Furthermore, using the Williamson–Hall method from XRD diffraction peaks,^30^ the crystallite size values for samples Zr_46_Si_2_N_52_, Zr_39_Si_8_N_53__300C, and Zr_39_Si_8_N_53__500C were estimated: 15.9, 4.2, and 5.4 nm, respectively. Since Zr_39_Si_8_N_53_ exhibits amorphous behavior, it was not possible to evaluate the crystallite size for this sample.

Finally, there is a clear reduction in crystallite size values upon comparison of Zr_46_Si_2_N_52_ with Zr_39_Si_8_N_53__300C and Zr_39_Si_8_N_53__500C samples. This behavior can be explained by the higher silicon content in these samples, which promotes the formation of more Si_*x*_N_*y*_ clusters. These compounds act as barriers to ZrN growth, leading to grain refinement in these samples.^31–33^

Aiming to promote a complementary discussion about how the transition from amorphous to crystalline structure influences sample morphology, we show SEM-FEG cross section micrographs of all deposited coatings/samples in Figure 3.

**Figure 3:**
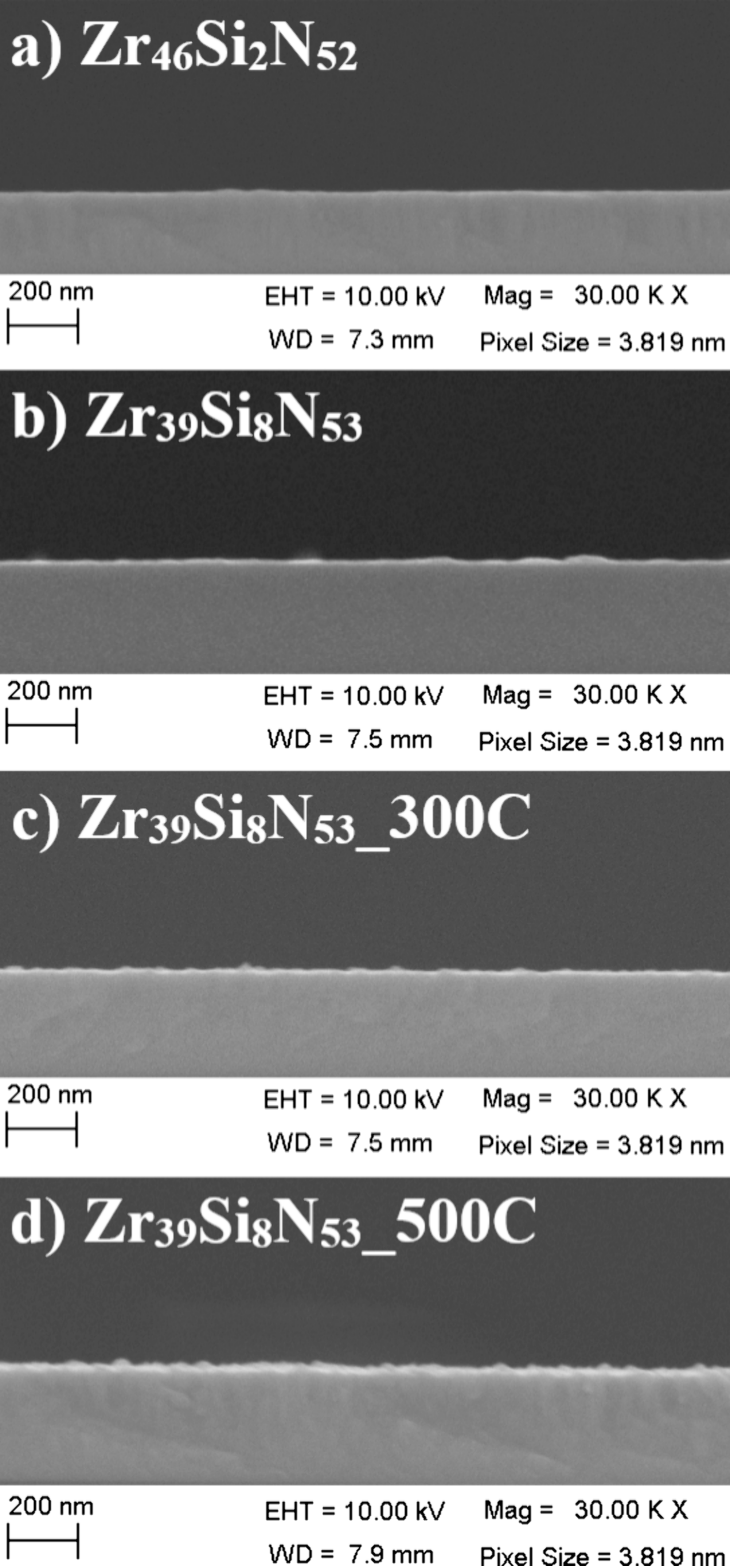
Cross-section SEM-FEG images for samples (a) Zr_46_Si_2_N_52_, (b) Zr_39_Si_8_N_53_, (c) Zr_39_Si_8_N_53__300C, and (d) Zr_39_Si_8_N_53__500C.

Sample Zr_46_Si_2_N_52_ (Figure 3a) exhibits a columnar morphology with elongated grains, a common structure observed in thin films with (111) preferential orientation, once this texture grows perpendicularly to the substrate plane.^34–36^ The columnar structure can be identified by the vertical shading observed in certain regions, indicating the presence of voids between columns, a typical characteristic of columnar growth in thin films.^37,38^ In contrast, sample Zr_39_Si_8_N_53_ (Figure 3b) presents a dense structure, without columnar growing, which is typical of amorphous samples.^39^

Despite the crystallization observed in the XRD pattern for Zr_39_Si_8_N_53__300C, no significant morphological changes were observed in this sample (Figure 3c) when compared to amorphous Zr_39_Si_8_N_53_ (Figure 3b). The high silicon content in sample Zr_39_Si_8_N_53__300C (forming large Si_*x*_N_*y*_ clusters) combined with substrate heating at 300 °C produces a grain refinement of the ZrN phase, as observed in XRD results, suggesting a densification of the microstructure and suppression of column formation.

On the other hand, Zr_39_Si_8_N_53__500C (Figure 3d) exhibited a morphology similar to that observed for sample Zr_46_Si_2_N_52_ (Figure 3a). The higher thermal energy provided during deposition at 500 °C resulted in a slight increase in grain size in comparison to sample Zr_39_Si_8_N_53__300C (4.2 to 5.4 nm). This increase was sufficient to promote better structural organization, with the return of the columnar growth. Overall, despite the presence (or not) of a columnar structure, it was not possible to identify relevant differences in morphology by the FEG-SEM analyses among the samples.

The results observed in XRD and FEG-SEM for samples Zr_39_Si_8_N_53__300C and Zr_39_Si_8_N_53__500C suggest possible modifications in mechanical and thermal properties, which will be discussed in the following sections.

### Nanohardness

Hardness results for samples Zr_46_Si_2_N_52_, Zr_39_Si_8_N_53_, Zr_39_Si_8_N_53__300C, and Zr_39_Si_8_N_53__500C are presented in Figure 4.

**Figure 4:**
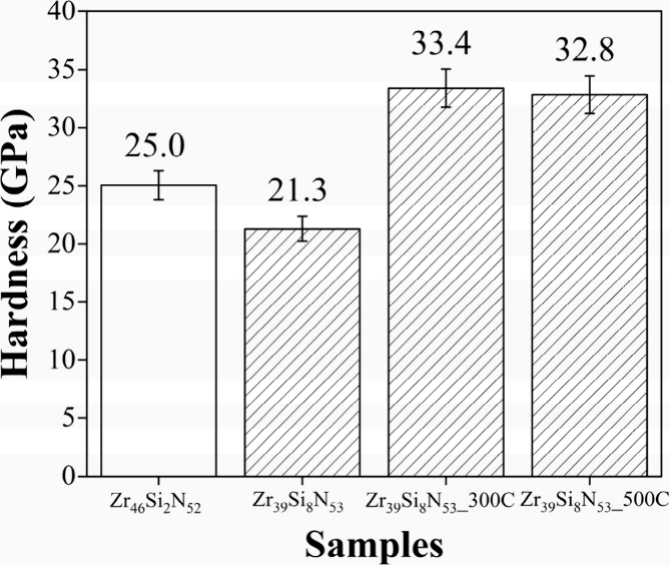
Hardness values for Zr_46_Si_2_N_52_, Zr_39_Si_8_N_53_, Zr_39_Si_8_N_53__300C, and Zr_39_Si_8_N_53__500C samples.

The Zr_39_Si_8_N_53_ sample exhibits a hardness reduction of more than 15% compared to Zr_46_Si_2_N_52_. As previously mentioned, this behavior was already expected, given that higher silicon content in Zr–Si–N thin films led to a decline in their mechanical performance. However, despite containing 8 at. % Si in their structure, both Zr_39_Si_8_N_53__300C and Zr_39_Si_8_N_53__500C present a hardness increase exceeding 30% in comparison to Zr_46_Si_2_N_52_, confirming that the crystallization process has a direct influence on enhancing this mechanical property.

Two factors need to be considered to explain the hardness increase observed in samples Zr_39_Si_8_N_53__300C and Zr_39_Si_8_N_53__500C: (a) XRD analyses revealed structural reorganization in the coatings with the development of grains with orientation (200). Since these oriented grains possess lower surface energy and fewer defects when compared to (111) planes, this transition is frequently beneficial to improve the mechanical properties of the coatings, as it results in a more stable system and leads to an increase in bonding strength.^36,40,41^ Consequently, coatings with (200) grains oriented tend to exhibit enhanced hardness, as earlier observed in other systems.^21,42,43^ (b) The high silicon content promotes the formation of large Si_*x*_N_*y*_ clusters and a significant refinement of ZrN grains, as observed in XRD diffractograms, where smaller crystallite size was found for samples Zr_39_Si_8_N_53__300C and Zr_39_Si_8_N_53__500C when compared to the sample Zr_46_Si_2_N_52_.

It is well established that grain refinement enhances the resistance to dislocation movement due to the increased grain boundaries density, contributing to higher hardness values.^44,45^ However, excessive grain refinement also increases the grain boundary area, where atomic bonding forces are weaker, which probably occurs in the amorphous sample Zr_39_Si_8_N_53_. This structural characteristic may facilitate grain rotation or deformation when stress is applied, which explains the low hardness value observed for this sample. Similar behavior has been reported in other ternary nitride thin films systems.^46,47^ In consonance to literature, all these factors observed in GIXRD patterns individually and collectively contribute positively to the enhanced hardness of the thin films.

### Oxidation Resistance

In order to investigate the influence of crystallization on the high temperature oxidation resistance of Zr–Si–N thin films, all deposited samples were subjected to tests at 600 °C. This temperature was selected because it is above the reported oxidation temperature of ZrN.^48,49^

Figure 5a shows the diffraction patterns for samples Zr_46_Si_2_N_52_ and Zr_39_Si_8_N_53_. Only high-intensity peaks associated with monoclinic and tetragonal phases of zirconium oxide (ZrO_2_) (PDF-65-1023 and PDF-7-1282) could be identified for sample Zr_46_Si_2_N_52_.

**Figure 5:**
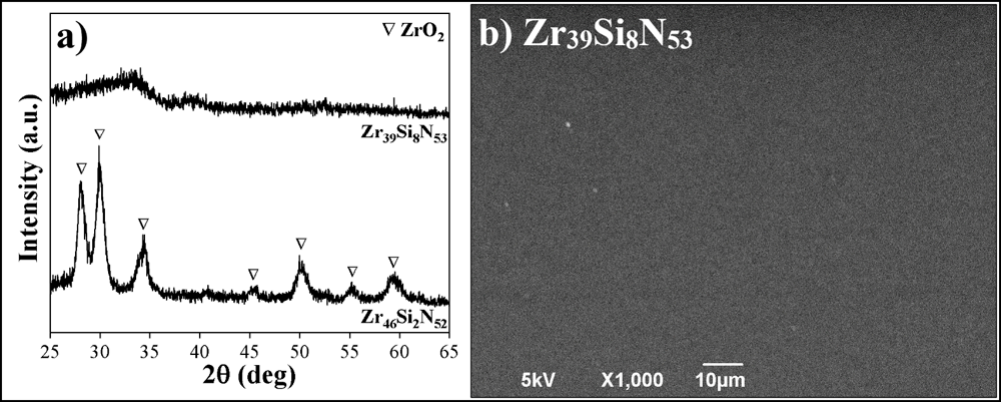
(a) GIXRD analyses for samples Zr_46_Si_2_N_52_ and Zr_39_Si_8_N_53_ after oxidation tests at 600 °C,. (b) SEM image for oxidized sample Zr_39_Si_8_N_53_.

There is no evidence of ZrN peaks, indicating the complete oxidation of the coatings at such a temperature. This behavior is expected given that this thin film exhibited a columnar grain structure, which facilitates oxygen diffusion and consequently results in low thermal resistance.^50^

Zr_39_Si_8_N_53_ after the oxidation test presented the same amorphous behavior as observed in the as-deposited sample. As observed in Figure 3b, this sample shows a dense structure with no visible columnar growth, which could act as a barrier to oxygen diffusion, preventing its penetration.

To confirm whether sample Zr_39_Si_8_N_53_ has resisted oxidation, a SEM surface analysis was conducted, as shown in Figure 5b. The result is very similar to that observed for as deposited samples, without bubbles or thin film delamination with substrate exposure, suggesting that the sample resists the oxidation at 600 °C.

GIXRD patterns for the crystalline samples Zr_39_Si_8_N_53__300C and Zr_39_Si_8_N_53__500C are presented in Figure 6a. Both samples exhibit peaks related to ZrO_2_; however, ZrN peaks were preserved, suggesting a noticeable improvement in oxidation resistance in comparison with Zr_46_Si_2_N_52_. Nevertheless, samples still failed to fully resist oxidation at 600 °C.

**Figure 6:**
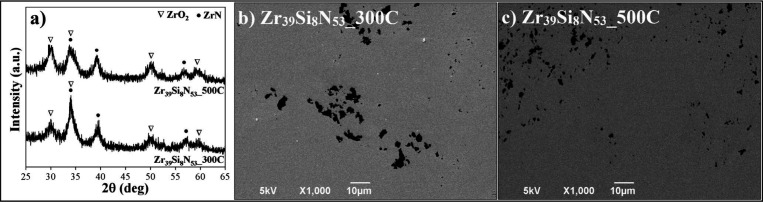
(a) GIXRD analyses for samples Zr_39_Si_8_N_53__300C and Zr_39_Si_8_N_53__500C after oxidation tests at 600 °C. SEM images for oxidized samples (b) Zr_39_Si_8_N_53__300C and (c) Zr_39_Si_8_N_53__500C.

Figure 6b,c shows SEM analyses for samples Zr_39_Si_8_N_53__300C and Zr_39_Si_8_N_53__500C, respectively. Consistent with the GIXRD patterns, both samples did not resist the oxidation test at 600 °C, exhibiting spallation and substrate exposure.

The oxidation tests performed on the Zr–Si–N samples indicate that neither the preferential growth orientation (111) or (200) nor the presence of prominent columnar growth or dense morphology, had a significant influence on their thermal stability.

The silicon content (forming large Si_*x*_N_*y*_ clusters), combined with an amorphous structure, is the primary factor enhancing the oxidation resistance of zirconium silicon nitride thin films. This is because crystallinity in thin films tends to facilitate oxygen diffusion into the matrix due to the ordered atomic arrangement and the presence of grain boundaries, which act as diffusion pathways and accelerate coating degradation. In contrast, the amorphous structure, characterized by atomic disorder and the absence of grain boundaries, impedes oxygen diffusion within the coatings, thereby enhancing the oxidation resistance.

## Conclusions

Amorphous and crystalline Zr–Si–N thin films containing 8 at. % Si were successfully deposited by reactive magnetron sputtering to investigate which is the dominant factor, crystallinity or silicon content, responsible for the mechanical and oxidation properties of these coatings. Therefore, the following is concluded.

- GIXRD and SEM-FEG analyses revealed that crystallization of samples Zr_39_Si_8_N_53__300C and Zr_39_Si_8_N_53__500C led to the development of the (200) plane, a change in growth morphology (from dense at 300 °C to columnar at 500 °C), and a reduction in crystallite size from 15.9 nm (2 at. % Si) to 4.2 and 5.4 nm, respectively.
- Nanohardness analyses showed that crystalline samples with 8 at. % exhibited an increase of over 35% in hardness when compared to amorphous Zr_39_Si_8_N_53_ samples. This improvement is attributed to the development of the (200) plane, which promotes a more stable system and results in increased bonding strength. Furthermore, grain refinement increases the resistance to dislocation movement due to the higher density of grain boundaries, thereby contributing to improved hardness values, confirming the relevance of the crystallization to change in mechanical properties.
- The oxidation tests revealed that crystallization reduced the thermal stability. Neither the development of the (200) plane nor the presence of columnar growth or dense morphology influenced the thermal stability of the crystalline samples with 8 at. % Si. Thus, it was concluded that a high silicon content, when associated with an amorphous structure, is the main factor responsible for enhancing the oxidation resistance of zirconium silicon nitride thin films. This improvement is attributed to the atomic disorder and the absence of grain boundaries, which hinder oxygen diffusion within the coatings and thereby increase their resistance to oxidation.

In summary, the results demonstrate that while crystallization significantly enhances the hardness of zirconium silicon nitride thin films, it does not provide the same benefit for their thermal performance.
